# Risk Factors and Quality of Life in Women with Urinary Incontinence in Kazakhstan: A Multicenter Case–Control Study

**DOI:** 10.3390/ijerph23070893

**Published:** 2026-07-10

**Authors:** Zhanylsyn Ryspayeva, Zaytuna Khismetova, Dinara Serikova-Esengeldina, Khalida Sharipova, Zaituna G. Khamidullina, Gulnara Khudaykulova, Yevgeniya Rahanskaya, Dana Kozhakhmetova, Kamila Akhmetova, Assiya Kussainova, Laura Kassym

**Affiliations:** 1 Department of Public Health, Non-Profit Joint Stock Company “Semey Medical University”, Semey 071400, Kazakhstan; zaituna.khismetova@smu.edu.kz; 2 Department of Obstetrics and Gynecology #1, Non-Profit Joint Stock Company “Astana Medical University”, Astana 010000, Kazakhstan; sharipova.kh@amu.kz (K.S.); hamidullina.z@amu.kz (Z.G.K.); 3 Department of Public Health & Healthcare Management #1, Tashkent State Medical University, Tashkent 100109, Uzbekistan; gkhudaykulova@gmail.com; 4 Department of Traumatology and Pediatric Surgery, Non-Profit Joint Stock Company “Semey Medical University”, Semey 071400, Kazakhstan; evgeniya.rahanskaya@smu.edu.kz; 5 Department of Internal Medicine and Rheumatology, Non-Profit Joint Stock Company “Semey Medical University”, Semey 071400, Kazakhstan; dana.kozhakhmetova@smu.edu.kz; 6 Department of Public Health and Management, Non-Profit Joint Stock Company “Astana Medical University”, Astana 010000, Kazakhstan; akhmetova.km@amu.kz; 7 Department of General Medical Practice with a Course of Evidence-Based Medicine, Non-Profit Joint Stock Company “Astana Medical University”, Astana 010000, Kazakhstan; kussainova.as@amu.kz (A.K.); kassym.l@amu.kz (L.K.)

**Keywords:** urinary incontinence, women’s health, risk factors, quality of life, Kazakhstan

## Abstract

**Highlights:**

**Public health relevance—How does this work relate to a public health issue?**

- Urinary incontinence represents an underrecognized women’s health problem in Kazakhstan, with important clinical and public health implications.
- The study highlights the burden of UI beyond clinical symptoms, including reduced quality of life and greater impairment among women with urgency urinary incontinence.

**Public health significance—Why is this work of significance to public health?**

- This study provides population-specific evidence from Kazakhstan, where data on UI risk factors and quality-of-life impact remain limited.
- Obstetric and metabolic factors, including vaginal delivery, multiple birth, macrosomia, and overweight or obesity, were independently associated with UI.

**Public health implications—What are the key implications or messages for practitioners, policy makers and/or researchers in public health?**

- Prevention strategies should integrate weight management, antenatal counseling, postpartum follow-up, and early assessment of pelvic floor dysfunction.
- Further research is needed to improve UI screening, reduce stigma, and develop culturally appropriate public health interventions for women in Kazakhstan.

**Abstract:**

Background: Urinary incontinence (UI) is a common condition that affects women’s physical, psychological, and social well-being. Evidence from Central Asia remains limited. This study assessed factors associated with UI, its subtypes, and quality-of-life impairment among women in Kazakhstan. Methods: A multicenter age-matched case–control study was conducted from February to May 2025 in healthcare facilities across Kazakhstan. The study included 2061 women. Sociodemographic, obstetric, lifestyle, comorbidity, and symptom data were collected using a structured questionnaire. Quality of life was assessed using Kazakh and Russian versions of the Incontinence Quality of Life Questionnaire (I-QOL). Multivariable logistic regression with backward elimination identified factors independently associated with UI. Results: UI was identified in 687 women (33.3%). Stress urinary incontinence (SUI) was the most common subtype (*n* = 356; 51.8%), followed by urgency urinary incontinence (UUI) (*n* = 191; 27.8%) and mixed urinary incontinence (MUI) (*n* = 140; 20.4%). UI was independently associated with vaginal delivery (OR = 1.48), multiple birth (OR = 2.26), macrosomia (OR = 1.83), and BMI ≥ 25 kg/m^2^ (OR = 2.08). UUI showed the greatest burden, including the lowest total I-QOL score [36.4 (23.9–88.6)]. Conclusions: UI in Kazakhstan was mainly associated with obstetric and metabolic factors, supporting targeted prevention through weight management, antenatal care, and early pelvic floor assessment.

## 1. Introduction

Urinary incontinence (UI) is one of the most common pelvic floor disorders among women [1] and is defined as any involuntary leakage of urine [2]. In clinical practice, three main types of UI are generally distinguished: stress urinary incontinence (SUI), which occurs during physical exertion, coughing, or sneezing; urgency urinary incontinence (UUI), which is associated with a sudden and difficult-to-defer urge to void; and mixed urinary incontinence (MUI), which combines features of both stress and urgency forms [2]. International studies indicate that the prevalence of UI among women varies considerably depending on the age of the study population, diagnostic criteria used, study methodology, and patterns of healthcare-seeking behavior [3]. In the United States, projected estimates suggest that the number of women affected by UI may rise from 18.3 million in 2010 to 28.4 million by 2050, reflecting the growing healthcare burden of this condition [4]. High prevalence rates have also been reported in European studies, reaching 48.3% in Germany and 46.4% in Denmark [5]. In Asian countries, the reported prevalence of UI among women ranges approximately from 24% to 46% [6,7].

Risk factors for UI should be interpreted in light of the clinical and pathogenetic heterogeneity of this condition [8,9]. In the study by Wang et al. (2023), which included 5800 women, the prevalence of UI increased with advancing age and higher body mass index (BMI), while SUI was identified as the most common subtype [8]. According to Abufaraj et al. (2021), based on data from the National Health and Nutrition Examination Survey for 2005–2018, obesity, smoking, comorbid conditions, and postmenopausal hormone therapy were associated with all types of UI [9]. At the same time, evidence from different regions suggests that the pattern of risk factors may vary across populations. For example, the systematic review by Hammad (2021), which focused on Gulf countries, reported substantial heterogeneity across studies, with chronic respiratory diseases and constipation most frequently identified as major predisposing factors [10]. These findings highlight the need for population-specific assessment of UI risk factors in each setting.

The coexistence of multiple risk factors may aggravate the course of UI and intensify its impact on women’s physical and psychological well-being [1]. UI symptoms are frequently associated with restrictions in daily and physical activities, social withdrawal, anxiety, reduced self-esteem, and impaired sexuality [11]. A substantial proportion of women, particularly in Muslim-majority countries, do not seek medical care because of embarrassment and the perception of UI as a natural or incurable condition [10]. This issue is of particular relevance for Kazakhstan, where evidence on UI and its effect on women’s quality of life remains limited. Available publications mainly focus on specific subgroups and do not provide a comprehensive assessment of the true burden of UI in the general female population [12]. The problem may also be underestimated because of cultural barriers, limited awareness, and low healthcare-seeking behavior [13,14]. In addition, the region has certain reproductive characteristics, including the high social and demographic importance of multiparity [15], short interpregnancy intervals, and the birth of infants with high birth weight, which may further increase the relevance of studying UI in this population.

Based on these considerations, the primary aim of this study was to identify factors associated with UI among women in Kazakhstan. The secondary aim was to evaluate clinical characteristics and quality of life according to UI subtype.

## 2. Materials and Methods

### 2.1. Study Design and Settings

To investigate risk factors associated with UI, we conducted a multicenter, age-matched case–control study among women attending medical institutions providing specialized UI care across Kazakhstan between February and May 2025. The study included two groups of participants. The case group comprised women diagnosed with UI by qualified specialists. The presence of UI among case participants and its absence among control participants were established using the diagnostic criteria specified in the national clinical protocol Stress Urinary Incontinence in Women and the NICE quality standard Urinary Incontinence in Women [16,17]. Eligible participants in both groups were women aged ≥18 years who provided written informed consent. Exclusion criteria were: (i) pregnancy; (ii) acute urinary tract infection; (iii) advanced pelvic organ prolapse; (iv) neurological disorders affecting bladder function; and (v) cognitive impairment precluding informed consent or questionnaire completion. The reporting of this study followed the STROBE guidelines for case–control studies, and the completed checklist is provided in Appendix A.

The control group consisted of women without UI who were age-matched to cases within ±2 years at a 1:2 ratio [18]. Cases and controls were frequency matched by age to achieve comparable age distributions between the two groups. Age was controlled for by the matching procedure, and its distribution was confirmed to be comparable between groups. The control group included women seeking care at the same healthcare facilities for urogynecological conditions other than UI. All matched cases were examined by the same specialists, and UI was excluded during the clinical assessment.

### 2.2. Data Collection and Training

**Figure 1:**
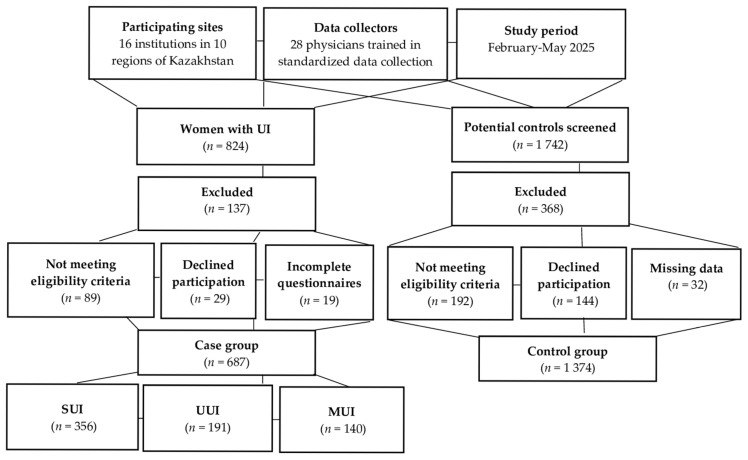
Flow diagram of participant recruitment and selection for the case–control study. UI-urinary incontinence; SUI-stress urinary incontinence; UUI-urgency urinary incontinence; MUI-mixed urinary incontinence.

The study was conducted in healthcare facilities providing urological and gynecological care, including services for women with UI, such as outpatient clinics, family medicine practices, private clinics, and specialized medical offices. An invitation letter describing the study was distributed through professional community groups across Kazakhstan, including gynecologists, urologists, rehabilitation specialists, and other healthcare professionals involved in the management of patients with UI. Healthcare professionals who agreed to participate underwent a one-day online training session to ensure standardized patient recruitment procedures and appropriate assistance with questionnaire completion. Standardized recruitment procedures were ensured by the principal investigator (Z.R.) and the study coordinator (D.S.-E.). Regular communication with participating sites was maintained throughout the study to ensure adherence to the standardized recruitment process. Finally, 28 physicians from 16 institutions across 10 cities and regions of Kazakhstan participated in data collection. They assisted participants in both the case and control groups in completing the digital questionnaire. All records were carefully screened, and entries containing technical errors or inappropriate responses were excluded from the analysis. Figure 1 illustrates the participant recruitment process and selection of the final study sample.

### 2.3. Research Tools

To assess the impact of UI on quality of life, translated and culturally adapted Russian- and Kazakh-language versions of the Incontinence Quality of Life Questionnaire (I-QOL) were used. The I-QOL is a disease-specific instrument designed to evaluate the effect of UI on patients’ quality of life. It comprises 22 items across three domains: avoidance and limiting behavior (ALB), psychosocial impact (PS), and social embarrassment (SE). Each item is scored using a 5-point Likert scale, with higher scores indicating better quality of life and a lower negative impact of UI [19]. The questionnaire was translated from English into Kazakh and Russian using a forward–backward translation procedure. Initially, two independent bilingual experts translated the original English version into Kazakh and Russian. The translated version was subsequently back-translated into English by an independent translator blinded to the original questionnaire. Discrepancies were reviewed and resolved by the research team to ensure semantic and conceptual equivalence. The final Kazakh and Russian versions were pilot-tested for clarity and comprehensibility prior to data collection. Internal consistency reliability of the translated questionnaire was assessed using Cronbach’s alpha coefficient, which demonstrated acceptable reliability (Cronbach’s α > 0.70 for the Kazakh version and >0.80 for the Russian version).

Additionally, the principal investigators developed supplementary questionnaire sections covering: (i) sociodemographic characteristics, including age, place of residence, marital status, educational level, and sexual activity; (ii) potential risk factors, such as obstetric history, BMI, heavy physical workload, and comorbidities; and (iii) self-reported UI characteristics, including symptom duration, severity, and frequency. Sections (i) and (ii) were administered to all participants, whereas sections (iii) and the I-QOL questionnaire were completed only by participants diagnosed with UI.

### 2.4. Definitions Used

To assess potential risk factors for UI, participants were asked about the presence of relevant medical and lifestyle-related conditions. During a one-day online training session, specialists involved in questionnaire administration were instructed on the definitions and interpretation of the study terms to ensure consistency in data collection. Thus, macrosomia was defined as a birth weight of ≥4000 g, irrespective of gestational age [20]. Multiple birth was defined as an obstetric condition when a woman is carrying more than one baby, e.g., twins or triplets [21]. Heavy lifting was defined as regular lifting of objects weighing ≥10 kg during occupational or daily activities. BMI was calculated as weight in kilograms divided by height in meters squared (kg/m^2^). Participants were classified according to the World Health Organization (WHO) criteria as underweight (<18.5 kg/m^2^), normal weight (18.5–24.9 kg/m^2^), overweight (25.0–29.9 kg/m^2^), and obese (≥30.0 kg/m^2^) [22]. Menopause was defined as the permanent cessation of menstruation for 12 consecutive months in the absence of other pathological or physiological causes [23].

### 2.5. Ethical Aspects

Ethical approval was obtained from the Local Ethical Committee, Semey Medical University, Semey, Kazakhstan (Protocol #2, dated 5 December 2024) and the research was conducted in compliance with principles of the Declaration of Helsinki and the Guideline for Good Clinical Practice. All participants provided written informed consent.

### 2.6. Statistical Analysis

Statistical analyses were performed using IBM SPSS Statistics for Windows, Version 27.0 (IBM Corp., Armonk, NY, USA).

A priori sample size estimation was performed for a matched case–control study with a 1:2 case-to-control ratio, assuming a two-sided significance level of 0.05, 80% statistical power, and an expected moderate association between exposure and UI. The estimated minimum sample size was substantially lower than the final study population. Ultimately, the study included 687 matched cases and 1374 matched controls, providing sufficient statistical power for the evaluation of multiple risk factors.

Continuous variables were presented as median with the first and third quartiles (Q1–Q3) due to distributional asymmetry. Group differences were assessed using appropriate rank-based tests. Categorical variables were summarized as frequencies and percentages, with comparisons performed using the chi-square test or Fisher’s exact test when applicable.

Univariable logistic regression analyses were performed to estimate crude odds ratios (ORs) and corresponding 95% CIs for all potential risk factors. Variables demonstrating evidence of association in the univariable analysis (*p* < 0.20), together with variables considered clinically relevant based on previous evidence, were entered into the multivariable logistic regression model. A backward likelihood ratio elimination procedure was used to identify factors independently associated with UI. Adjusted odds ratios (aORs) with 95% confidence intervals are reported for the final model. A two-sided *p*-value < 0.05 was considered statistically significant.

## 3. Results

The study included 687 cases with UI and 1374 matched controls without UI. Table 1 presents the baseline sociodemographic characteristics of both groups. Among the women with UI, SUI was the predominant subtype (51.8%, *n* = 356), followed by UUI (27.8%, *n* = 191) and MUI (20.4%, *n* = 140).

Rural residence was more common among women with UI compared with those without UI (22.6% vs. 16.3%), whereas urban residence predominated in both groups, particularly among women without UI (74.5% vs. 69.1%) (*p* = 0.002). Women with UI less frequently had higher education (61.3% vs. 75.5%) and more often reported widowhood (7.9% vs. 2.2%) compared with women without UI (*p* < 0.001).

Regarding sexual activity, women with UI less frequently reported regular sexual activity several times per week (9.0% vs. 19.3%) or once weekly (4.4% vs. 14.3%), while sexual inactivity was more than twice as common among women with UI than among those without UI (23.0% vs. 10.9%, *p* < 0.001).

Table 2 presents the distribution of potential risk factors according to the presence of UI. Women with UI had significantly higher parity compared to those without UI, with grand multiparity (≥5 births) observed in 14.0% versus 5.5%, respectively (*p* < 0.001). Vaginal delivery was more common among women with UI (77.3% vs. 68.6%), whereas nulliparity was less frequent (6.3% vs. 15.7%) (*p* < 0.001). A history of multiple births was reported more frequently in the UI group (13.5% vs. 6.0%, *p* < 0.001). Similarly, macrosomia was substantially more prevalent among women with UI compared to non-UI participants (24.9% vs. 12.2%, *p* < 0.001).

BMI differed significantly between the groups. Obesity (BMI > 30 kg/m^2^) was observed in 18.8% of women with UI compared with 13.0% in the non-UI group, while normal BMI was less frequent among women with UI (32.8% vs. 52.1%) (*p* < 0.001). Heavy lifting was also more commonly reported in the UI group (81.7% vs. 69.4%, *p* < 0.001).

No statistically significant differences were identified between groups regarding diabetes (10.0% vs. 12.5%, *p* = 0.256), arterial hypertension (34.6% vs. 37.2%, *p* = 0.515), pelvic surgery (13.4% vs. 14.6%, *p* = 0.763), back pain (34.6% vs. 32.8%, *p* = 0.426), or menopause status (37.4% vs. 33.1%, *p* = 0.151). 

Additionally, the analysis of risk factor differences across UI subtypes was performed (Table S1). Significant differences were observed for parity, history of macrosomia, BMI, diabetes, arterial hypertension, pelvic surgery, back pain, and menopausal status (all *p* < 0.05). Women with MUI were more likely to have a history of ≥5 deliveries (25.0%) and macrosomia (48.6%) compared with those with SUI or UUI. Obesity (BMI ≥ 30 kg/m^2^) was most prevalent among women with MUI (30.7%), whereas overweight (BMI 25.0–29.9 kg/m^2^) predominated in the UUI group (51.8%). Diabetes and arterial hypertension were more frequently reported among women with UUI (13.1% and 40.8%, respectively), while a history of pelvic surgery was more common in the UUI (16.8%) and MUI (15.0%) groups than in the SUI group (11.0%). Back pain was reported most frequently by women with UUI (55.0%), and menopause was substantially more common among women with UUI (81.2%) and MUI (67.9%) than among those with SUI (46.6%). No significant differences were observed between UI subtypes with respect to mode of delivery, history of multiple birth, or heavy lifting (*p* > 0.05).

Table 3 presents the multivariable logistic regression analysis with backward elimination performed to identify factors independently associated with UI. Vaginal delivery (OR 1.48, 95% CI 1.19–1.85), multiple birth (OR 2.26, 95% CI 1.64–3.12), macrosomia (OR 1.83, 95% CI 1.42–2.34), and BMI ≥ 25 kg/m^2^ (OR 2.08, 95% CI 1.71–2.54) remained independently associated with UI (Nagelkerke R^2^ = 0.094; model likelihood ratio test: *p* < 0.001). Parity, heavy lifting, diabetes, hypertension, pelvic surgery, back pain, and menopause were excluded from the final model during backward elimination due to lack of statistical significance.

Table 4 summarizes the clinical characteristics according to UI subtype. Significant differences were observed in symptom duration, severity, and frequency of UI episodes among the groups. Women with UUI reported the longest symptom duration (36 (3.5–60) months vs. 24 (0–48) months in SUI and 24 (2–60) months in MUI; *p* = 0.035). High symptom severity was most prevalent in the UUI group (48.2%), compared with SUI (38.8%) and MUI (39.3%) (*p* = 0.024). Regarding symptom frequency, daily episodes were more common in women with UUI, with 36.1% reporting leakage 3–4 times per day versus 24.4% in SUI and 23.6% in MUI (*p* = 0.033).

Table 5 demonstrates markedly reduced quality-of-life scores across all domains of the I-QOL questionnaire among women with UI. Women with SUI had the highest median quality-of-life scores, whereas the lowest scores were generally observed in the UUI group. Median ALB scores were 46.9 (25.0–93.8) in SUI, 37.5 (25.0–84.4) in UUI, and 37.5 (25.0–93.8) in MUI (*p* = 0.012). Similarly, PS scores differed significantly between groups, with median values of 44.4 (25.0–100.0), 36.1 (25.0–91.7), and 41.7 (25.0–100.0) in SUI, UUI, and MUI, respectively (*p* = 0.009). For the SE domain, women with SUI and MUI had comparable median scores, both higher than those observed in the UUI group: 40.0 (25.0–95.0), 35.0 (25.0–85.0), and 40.0 (25.0–95.0), respectively (*p* = 0.019). The overall I-QOL total score also differed significantly across UI subtypes, with the highest median score in SUI [43.2 (26.1–95.5)] and the lowest in UUI [36.4 (23.9–88.6)], while women with MUI demonstrated intermediate values [37.5 (25.0–96.1)] (*p* = 0.009). In the overall cohort of women with UI, the median standardized I-QOL scores indicated substantial impairment in quality of life across all domains. Median (Q1–Q3) scores were 40.6 (25.0–90.6) for ALB, 41.7 (25.0–97.2) for PS, 40.0 (25.0–95.0) for SE, and 39.8 (25.0–94.3) for the overall I-QOL score.

## 4. Discussion

### 4.1. Risk Factors for UI in the Kazakhstani Female Population

According to the findings of the present study, several groups of women in the Kazakhstani population can be identified as having a higher risk of UI.

First, rural residence was statistically significantly associated with UI symptoms. In the Kazakhstani context, this pattern may be related to more demanding living conditions in rural settings, including higher levels of daily physical workload and limited access to specialized medical care. This interpretation is consistent with previous evidence showing that women exposed to physically demanding work-related conditions, such as heavy lifting and prolonged awkward postures, may have an increased likelihood of UI [24]. By contrast, another study reported that UI symptoms were 1.6 times more common among women living in urban areas. The authors attributed this finding to the shorter life expectancy of rural women and the lower prevalence of overweight and obesity in rural populations [8].

Second, women with UI were less likely to have higher education and more likely to report widowhood. Comparable findings have been reported in a previous study, in which UI was more frequent among unemployed and retired women, as well as among widowed or separated women, with prevalence reaching 19.3%. The authors suggested that these associations may be explained by older age, the accumulation of chronic conditions, and differences in awareness and healthcare-seeking behavior [25].

In our study, sexual inactivity was reported almost twice as often among women with UI, which may reflect the combined influence of age, marital status, and the direct impact of symptoms on intimate life. In the study by Felippe et al. (2017), women with UI had lower levels of sexual desire, comfort, satisfaction, and partner harmony, which may be related to fear of urine leakage and reduced self-confidence [26]. At the same time, among nulliparous Australian women younger than 30 years, the highest rates of UI were observed in sexually active women [27].

Women with a significant obstetric and gynecological history, including high parity, vaginal delivery, multiple pregnancy/delivery, and macrosomia, had a higher risk of UI. These findings are consistent with global evidence indicating that repeated pregnancies and childbirth may weaken the musculo-fascial and neural structures of the pelvic floor, impair bladder and urethral support, and reduce the effectiveness of urethral closure mechanisms [28,29]. For example, Zhang et al. (2016) showed that SUI was more frequently reported during pregnancy (49.5%) and after childbirth (43.6%), whereas it was present in only 6.9% of women before pregnancy [30]. Another article demonstrated that vaginal delivery nearly doubled the risk of UI compared with cesarean section [31]. Fetal macrosomia appeared to be of particular importance in our study, being reported in 24.9% of women with UI compared with 12.2% of women without UI. These findings highlight macrosomia as a potentially relevant component of the obstetric risk profile in the Kazakhstani female population. In a recent systematic review and meta-analysis, data from 18 studies involving 30,070 women showed that birthweight >4000 g was also associated with a higher risk of postpartum UI (OR 1.49; 95% CI: 1.24–1.80) [32]. However, these findings are not consistent across other population-based samples. For instance, Zhu et al. (2023) reported conflicting findings in the Chinese population [33], whereas a large Italian study found no association between macrosomia and an increased risk of uterine prolapse [34]. Among all UI subtypes, obstetric history appeared to play a particularly important role in MUI, as women with MUI more often reported high parity and macrosomia. This is consistent with Wang et al., who identified multiple vaginal deliveries and macrosomia as independent risk factors for MUI in a large multicenter study of parous women [35].

An important group comprised women with metabolic disorders, among whom obesity and high BMI were significantly more common in respondents with UI. Higher BMI was also associated with UI subtype distribution, with obesity most frequent in MUI and overweight predominating in UUI. Sun et al. (2022) reported that each 1 kg/m^2^ increase in BMI was associated with a 7% higher likelihood of urinary incontinence, possibly due to increased intra-abdominal pressure [36]. This issue is particularly relevant for Kazakhstan, where, according to the Global Nutrition Report, the prevalence of obesity among adult women reaches 25.3% [37].

According to our findings, diabetes mellitus, arterial hypertension, back pain, pelvic surgery, and menopausal status were not statistically significantly associated with UI symptoms. This may suggest that, in the present sample, their contribution was less pronounced than that of factors more directly related to pelvic floor loading. Similarly, in a nationally representative sample of women in the United States, biochemical markers of diabetes were associated with stress and urgency UI only in unadjusted models, whereas these associations were no longer statistically significant after adjustment for BMI [38]. In a Korean national study, diabetes mellitus and arterial hypertension were also not identified as independent factors associated with UI, while age, BMI, and marital status remained the main correlates [39]. However, subtype-specific analysis showed significant differences for several of these factors. Diabetes mellitus, arterial hypertension, back pain, and menopausal status were more frequent among women with UUI, whereas pelvic surgery was more common in women with UUI and MUI than in those with SUI. This pattern is consistent with previous studies showing that UUI and MUI are more strongly related to older age, functional limitations, metabolic comorbidity, and higher BMI, while SUI is more closely linked to mechanical and obstetric factors [40,41].

### 4.2. Quality of Life and Health Policy Priorities in Women with UI

In the study sample, UUI was associated with the most unfavorable clinical course and the greatest reduction in quality of life. Women in this group had a longer duration of symptoms, a higher proportion of severe UI, more frequent daily leakage episodes, and the lowest scores across all I-QOL domains and the total I-QOL score. These findings are partly consistent with the review by Riss and Kargl (2011), which emphasized that urgency and UUI may have a greater impact on quality of life than SUI, as leakage episodes are less predictable and more difficult to control [42]. At the same time, our findings differ from several international studies. Frick et al. reported that MUI was associated with a more pronounced decline in quality of life among middle-aged and older women [43]. Similarly, Minassian et al. found that MUI was the most severe and bothersome subtype [44]. The pattern observed in our study may be explained by delayed healthcare-seeking, a higher comorbidity burden in women with UUI, and insufficient early correction of symptoms.

UI imposes a substantial burden on women’s physical, mental, and social well-being. It restricts daily activities, reduces quality of life, and is often accompanied by anxiety, social withdrawal, and depression [45]. The perception of UI symptoms as a “normal” consequence of childbirth or ageing, limited awareness of available treatment options, stigma, and embarrassment further aggravate the problem. These factors may delay healthcare-seeking and contribute to symptom persistence [46]. A recent study among young women in Kazakhstan showed that UI and overactive bladder symptoms are already present at a young age. This finding highlights the need for early detection of UI, including among younger women [12].

Given the risk profile identified in our study, healthcare measures should be targeted rather than universal. Screening for UI symptoms should be integrated into primary healthcare, routine obstetric and gynecological visits, postpartum follow-up, and counselling for peri- and postmenopausal women. For women with high parity, previous vaginal delivery, fetal macrosomia, or multiple pregnancy, priority should be given to early assessment of pelvic floor function and training in pelvic floor muscle exercises. In women with obesity, weight management and correction of metabolic risk factors are particularly important. In the presence of urgency symptoms, early detection of overactive bladder, behavioral interventions, and timely referral to specialized care are needed. This approach is consistent with NICE recommendations, which emphasize raising women’s awareness, early identification of pelvic floor dysfunction symptoms, and the use of non-invasive interventions at the primary care level [47]. For Kazakhstan, this implies a shift from a predominantly treatment-oriented model to a preventive strategy. Such a strategy should focus on risk groups, stigma reduction, and improved understanding that UI is not an inevitable consequence of childbirth or ageing, but a condition that can be corrected at an early stage.

### 4.3. Limitations

This study has several limitations. First, although the case–control design allowed us to assess associations between UI and potential risk factors, it did not permit causal inference or determination of the temporal sequence between exposures and symptom development. Therefore, the observed associations should be interpreted as potential relationships rather than evidence of direct causality.

Second, data on UI symptoms, obstetric history, comorbidities, and lifestyle-related factors were collected using a questionnaire, which may have introduced recall or reporting bias, particularly for sensitive issues such as urine leakage and sexual activity. Some clinical variables were also based on self-report, which may have affected the accuracy of factor classification.

Third, despite the inclusion of women from different healthcare settings and regions of Kazakhstan, the sample may not fully represent the general female population. Women attending healthcare facilities may differ from non-attenders in health status, symptom awareness, socioeconomic characteristics, and access to care.

As the questionnaires were administered with the involvement of several healthcare professionals after a one-day online training session, inter-assessor variability may have occurred and influenced the results.

Cases and controls were matched only by age; therefore, residual confounding by socioeconomic status and parity cannot be excluded.

Another limitation relates to the strategy used for multivariable model development. Although variable selection was informed by both univariable analyses and clinical relevance, the final model was derived using a backward elimination procedure. Consequently, alternative model-building approaches based on prespecified clinical and epidemiological knowledge may have produced slightly different estimates. Therefore, the adjusted associations should be interpreted with appropriate caution and confirmed in future studies.

Finally, quality of life was assessed at a single time point, limiting the ability to evaluate changes in symptom burden, disease progression, or the effect of treatment-seeking behavior over time.

## 5. Conclusions

UI represents a relevant and underrecognized women’s health issue in Kazakhstan. The findings indicate that its occurrence is shaped by a combination of sociodemographic, obstetric, metabolic, and clinical factors, while its impact on quality of life differs across UI subtypes. These results support the need to move beyond symptom-based care toward earlier identification, risk-oriented prevention, and routine integration of pelvic floor health into women’s healthcare services. Further longitudinal and population-based studies are needed to clarify causal pathways, assess regional differences, and evaluate the effectiveness of targeted preventive interventions.

## Figures and Tables

**Table 1:** Baseline characteristics of participants stratified by the presence of UI.

	Matched Controls (*n* = 1374)	Cases (*n* = 687)	*p*-Value
Age, years (Me (Q1–Q3))	51 (44–61)	52 (44–61)	0.517
Residence Type (*n*, %)			0.002
City	1023 (74.5%)	475 (69.1%)	
Small town	127 (9.2%)	57 (8.3%)	
Rural settlement	224 (16.3%)	155 (22.6%)	
Education (*n*, %)			<0.001
High school	50 (3.6%)	29 (4.2%)	
College	287 (20.9%)	237 (34.5%)	
Higher education	1037 (75.5%)	421 (61.3%)	
Marital status (*n*, %)			<0.001
Single	138 (10.0%)	40 (5.8%)	
Has a partner	98 (7.1%)	49 (7.1%)	
Married	1010 (73.5%)	494 (71.9%)	
Divorced	98 (7.1%)	50 (7.3%)	
Widow	30 (2.2%)	54 (7.9%)	
Sexual activity (*n*, %)			<0.001
Several times a week	265 (19.3%)	62 (9.0%)	
Once a week	196 (14.3%)	30 (4.4%)	
Several times a month	327 (23.8%)	115 (16.7%)	
Less than once a month	174 (12.7%)	132 (19.2%)	
Occasionally, during the year	130 (9.5%)	103 (15.0%)	
I am not sexually active	150 (10.9%)	158 (23.0%)	
I prefer not to answer	132 (9.6%)	87 (12.7%)	

Me—median, Q1—1st quartile, Q3—3rd quartile.

**Table 2:** Risk factors stratified by the presence of UI.

	Matched Controls (*n* = 1374)	Cases UI (*n* = 687)	*p*-Value
Parity (*n*, %)			<0.001
0	216 (15.7%)	43 (6.3%)	
1–2	676 (49.2%)	264 (38.4%)	
3–4	406 (29.5%)	284 (41.3%)	
≥5	76 (5.5%)	96 (14.0%)	
Delivery mode (*n*, %)			<0.001
Vaginal delivery	943 (68.6%)	531 (77.3%)	
Cesarean section	129 (9.4%)	50 (7.3%)	
Nulliparous (no history of childbirth)	216 (15.7%)	43 (6.3%)	
Mixed (vaginal and cesarean deliveries)	86 (6.3%)	63 (9.2%)	
Multiple birth (*n*, %)			<0.001
Yes	82 (6.0%)	93 (13.5%)	
No	1076 (78.3%)	551 (80.2%)	
Nulliparous (no history of childbirth)	216 (15.7%)	43 (6.3%)	
Macrosomia (*n*, %)			<0.001
Yes	168 (12.2%)	171 (24.9%)	
No	989 (72.0%)	473 (68.9%)	
Nulliparous (no history of childbirth)	217 (15.8%)	43 (6.3%)	
BMI (*n*, %)			<0.001
<18.5	40 (2.9%)	12 (1.7%)	
18.5–24.9	716 (52.1%)	225 (32.8%)	
25.0–29.9	439 (32.0%)	321 (46.7%)	
>30.0	179 (13.0%)	129 (18.8%)	
Heavy lifting (*n*, %)			<0.001
Yes	954 (69.4%)	561 (81.7%)	
No	355 (25.8%)	107 (15.6%)	
Not sure	65 (4.7%)	19 (2.8%)	
Diabetes (*n*, %)			0.256
Yes	172 (12.5%)	69 (10.0%)	
No	1158 (84.3%)	596 (86.8%)	
Not sure	44 (3.2%)	22 (3.2%)	
Arterial hypertension (*n*, %)			0.515
Yes	511 (37.2%)	238 (34.6%)	
No	789 (57.4%)	412 (60.0%)	
Not sure	74 (5.4%)	37 (5.4%)	
Pelvic surgery (*n*, %)			0.763
Yes	200 (14.6%)	92 (13.4%)	
No	1146 (83.4%)	580 (84.4%)	
Not sure	28 (2.0%)	15 (2.2%)	
Back pain (*n*, %)			0.426
Yes	450 (32.8%)	238 (34.6%)	
No	891 (64.8%)	428 (62.3%)	
Not sure	33 (2.4%)	21 (3.1%)	
Menopause (*n*, %)			0.151
Yes	455 (33.1%)	257 (37.4%)	
No	891 (64.8%)	416 (60.6%)	
Not sure	28 (2.0%)	14 (2.0%)	

**Table 3:** Multivariable logistic regression analysis of risk factors.

Variable	Adjusted OR	95% CI	*p*-Value
Vaginal delivery	1.48	1.19–1.85	<0.001
Multiple birth	2.26	1.64–3.12	<0.001
Macrosomia	1.83	1.42–2.34	<0.001
BMI ≥ 25	2.08	1.71–2.54	<0.001

OR—odds ratio; 95% CI—95% confidence interval.

**Table 4:** Clinical characteristics stratified by urinary incontinence subtype.

	SUI (*n* = 356)	UUI (*n* = 191)	MUI (*n* = 140)	Total (*n* = 687)	*p*-Value
Symptom duration, months Me (Q1–Q3)	24 (0–48)	36 (3.5–60)	24 (2–60)	24 (1–60)	0.035
Severity (*n*, %)					0.024
Low	100 (28.1%)	30 (15.7%)	36 (25.7%)	166 (24.2%)	
Moderate	118 (33.1%)	69 (36.1%)	49 (35.0%)	236 (34.4%)	
High	138 (38.8%)	92 (48.2%)	55 (39.3%)	285 (41.5%)	
Frequency (*n*, %)					0.033
Never	108 (30.3%)	39 (20.4%)	34 (24.3%)	181 (26.3%)	
1–2 times per month	40 (11.2%)	11 (5.8%)	12 (8.6%)	63 (9.2%)	
Approximately once a week (4 times)	5 (1.4%)	4 (2.1%)	3 (2.1%)	12 (1.7%)	
2–3 times per week	6 (1.7%)	3 (1.6%)	6 (4.3%)	15 (2.2%)	
Approximately once a day	38 (10.7%)	20 (10.5%)	23 (16.4%)	81 (11.8%)	
1–2 times per day	62 (17.4%)	40 (20.9%)	27 (19.3%)	129 (18.8%)	
3–4 times per day	87 (24.4%)	69 (36.1%)	33 (23.6%)	189 (27.5%)	
≥5 times per day	10 (2.8%)	5 (2.6%)	2 (1.4%)	17 (2.5%)	

Me—median, Q1—1st quartile, Q3—3rd quartile.

**Table 5:** Quality of life stratified by urinary incontinence subtype.

	SUI (*n* = 356) Me (Q1–Q3)	UUI (*n* = 191) Me(Q1–Q3)	MUI (*n* = 140) Me (Q1–Q3)	All Patients with UI (*n* = 687) Me (Q1–Q3)	*p*-Value
ALB	46.9 (25.0–93.8)	37.5 (25.0–84.4)	37.5 (25.0–93.8)	40.6 (25.0–90.6)	0.012
PS	44.4 (25.0–100.0)	36.1 (25.0–91.7)	41.7 (25.0–100.0)	41.7 (25.0–97.2)	0.009
SE	40.0 (25.0–95.0)	35.0 (25.0–85.0)	40.0 (25.0–95.0)	40.0 (25.0–95.0)	0.019
I-QOL total	43.2 (26.1–95.5)	36.4 (23.9–88.6)	37.5 (25.0–96.1)	39.8 (25.0–94.3)	0.009

I-QOL—Incontinence Quality of Life Questionnaire; ALB—avoidance and limiting behavior; PS—psychosocial impact; SE—social embarrassment; Me—median, Q1—1st quartile, Q3—3rd quartile.

## Data Availability

The original data presented in the study are openly available at GitHub 3.5.5 at: https://github.com/laurakassym-a11y/Manuscript-Ryspayeva-Dataset.git, accessed on 19 June 2026.

## Supplementary Materials

The following supporting information can be downloaded at: https://www.mdpi.com/article/10.3390/ijerph23070893/s1, Table S1: Risk factors stratified by the UI subtype.

## Abbreviations

The following abbreviations are used in this manuscript:

UI	urinary incontinence
SUI	stress urinary incontinence
UUI	urgency urinary incontinence
MUI	mixed urinary incontinence
BMI	body mass index
SD	standard deviation
ANOVA	analysis of variance
I-QOL	Incontinence Quality of Life Questionnaire
ALB	avoidance and limiting behavior
PS	psychosocial impacts
SE	social embarrassment

## Appendix A. STROBE Statement—Checklist for Case–Control Studies

**Table A1:** STROBE checklist for the reporting of the case-control study on urinary incontinence among women in Kazakhstan.

Section	Item	STROBE Recommendation	Reported on Page/Line	Information Reported in the Manuscript/Response
Title and abstract	1(a)	Indicate the study’s design with a commonly used term in the title or the abstract.	Title; Abstract, lines 2–3; 47–48	The manuscript title and abstract identify the study as a multicenter case–control study.
	1(b)	Provide in the abstract an informative and balanced summary of what was performed and what was found.	Abstract, lines 43–61	The abstract summarizes the introduction, objective, multicenter case–control design, sample, main associated factors, I-QOL findings, and conclusions.
Introduction				
Background/rationale	2	Explain the scientific background and rationale for the investigation being reported.	Introduction, lines 64–110	The Introduction describes the burden of urinary incontinence, its effect on women’s quality of life, and the lack of comprehensive evidence from Kazakhstan.
Objectives	3	State specific objectives, including any prespecified hypotheses.	Introduction, lines 108–110	The primary aim of the study was to identify factors associated with UI among women in Kazakhstan. The secondary aim was to evaluate clinical characteristics and quality of life according to UI subtype.
Methods				
Study design	4	Present key elements of study design early in the paper.	Methods, Study design, lines 115–117	The study is described as a multicenter age-matched case–control study conducted in Kazakhstan.
Setting	5	Describe the setting, locations, and relevant dates, including periods of recruitment, exposure, follow-up, and data collection.	Methods, Study setting and recruitment, lines 115–117, 138–143, 149–150	The study was conducted from February to May 2025 in outpatient clinics, family medicine practices, private clinics, and medical offices across 10 cities/regions of Kazakhstan; 28 physicians from 16 institutions collected data.
Participants	6(a)	Give the eligibility criteria, and the sources and methods of case ascertainment and control selection. Give the rationale for the choice of cases and controls.	Methods, Participants, lines 122–133	Eligible participants were women aged ≥18 years who provided written informed consent. Cases were women diagnosed with UI by qualified specialists; controls were women without UI. Exclusion criteria included pregnancy, acute urinary tract infection, advanced pelvic organ prolapse, neurologic bladder disorders, and cognitive impairment.
	6(b)	For matched studies, give matching criteria and the number of controls per case.	Methods, Participants/Matching, lines 127–133, 551–552	Controls were age-matched to cases within +/−2 years at a 1:2 ratio. The limitation that only age was used for matching is acknowledged in the Discussion.
Variables	7	Clearly define all outcomes, exposures, predictors, potential confounders, and effect modifiers. Give diagnostic criteria, if applicable.	Methods, Variables and definitions, lines 214–220	UI status, UI subtype, sociodemographic variables, obstetric history, BMI, comorbidities, menopause, heavy lifting, macrosomia (≥4000 g), and quality-of-life domains were defined. Potential confounders included socioeconomic status and parity/delivery-related variables.
Data sources/measurement	8 *	For each variable of interest, give sources of data and details of methods of assessment. Describe comparability of assessment methods if there is more than one group.	Methods, Research tools/Data collection, lines 196–212	Data were collected using structured questionnaires and the I-QOL instrument. Russian and Kazakh versions were prepared using forward-backward translation, pilot testing, and reliability assessment. Questionnaire administration was standardized through a one-day online training for participating healthcare professionals.
Bias	9	Describe any efforts to address potential sources of bias.	Methods; Discussion, Limitations	Age matching, standardized data collection forms, training of healthcare professionals, and exclusion criteria were used to reduce bias.
Study size	10	Explain how the study size was arrived at.	Methods, Sample size, lines 246–251	A priori sample size estimation was performed for a matched case–control study with a 1:2 case-to-control ratio, assuming a two-sided significance level of 0.05, 80% statistical power, and an expected moderate association between exposure and UI. The estimated minimum sample size was substantially lower than the final study population. Ultimately, the study included 687 matched cases and 1374 matched controls, providing sufficient statistical power for the evaluation of multiple risk factors.
Quantitative variables	11	Explain how quantitative variables were handled in the analyses. If applicable, describe which groupings were chosen and why.	Methods, Statistical analysis	Continuous variables were summarized as medians and interquartile ranges. BMI was categorized according to WHO categories; macrosomia and heavy lifting were analyzed using prespecified thresholds.
Statistical methods	12(a)	Describe all statistical methods, including those used to control for confounding.	Methods, Statistical analysis, lines 243–267	Analyses were performed in SPSS v27. Continuous variables were compared using rank-based tests; categorical variables with chi-square/Fisher’s exact tests. Unadjusted and multivariable logistic regression models with ORs and 95% CIs were used to identify factors associated with UI.
	12(b)	Describe any methods used to examine subgroups and interactions.	Methods, Statistical analysis	Subtype analyses were conducted for SUI, UUI, and MUI, including risk-factor distribution and I-QOL domain scores.
	12(c)	Explain how missing data were addressed.	Methods, Data collection, Lines 151–153	All records were carefully screened, and entries containing technical errors or inappropriate responses were excluded from the analysis.
	12(d)	If applicable, explain how matching of cases and controls was addressed.	Methods, Study Design and Setting, Lines 126–129	Cases and controls were frequency matched by age to achieve comparable age distributions between the two groups. Age was controlled for by the matching procedure and its distribution was confirmed to be comparable between groups.
	12(e)	Describe any sensitivity analyses.	Methods, Statistical analysis	No sensitivity analysis was performed
Results				
Participants	13(a) *	Report numbers of individuals at each stage of study, e.g., potentially eligible, examined for eligibility, confirmed eligible, included, completing follow-up, and analysed.	Materials and Methods; Data collection and Training, Lines 153–161	A flowchart was added to show recruitment and derivation of the final sample. The final sample included 2061 women: 687 cases with UI and 1374 matched controls.
	13(b) *	Give reasons for non-participation at each stage.	Materials and Methods; Data collection and Training, Lines 153–161	Reasons for exclusion/non-inclusion should be shown in the flowchart, including technical errors, inappropriate responses, and exclusion criteria
	13(c) *	Consider use of a flow diagram.	Materials and Methods; Data collection and Training, Lines 153–161	The flowchart is present
Descriptive data	14(a) *	Give characteristics of study participants and information on exposures and potential confounders.	Results, Table 1 and Table 2	Table 1 and Table 2 present characteristics of cases and matched controls, including sociodemographic, obstetric, clinical, and lifestyle-related variables.
	14(b) *	Indicate number of participants with missing data for each variable of interest.	Materials and Methods; Data collection and Training, Lines 153–161	The flowchart demonstrates the number of participants with missing data
Outcome data	15 *	Report numbers in each exposure category, or summary measures of exposure.	Results, Table 1 and Table 2	Exposure/risk-factor distributions are reported for cases and matched controls and, where applicable, by UI subtype.
Main results	16(a)	Give unadjusted estimates and, if applicable, confounder-adjusted estimates and their precision. Make clear which confounders were adjusted for and why they were included.	Results	Crude ORs with 95% CIs were proceeded
	16(b)	Report category boundaries when continuous variables were categorized.	Methods; Results tables	Category boundaries are reported, including BMI categories, age groups if used, macrosomia ≥ 4000 g, and heavy lifting ≥ 10 kg.
	16(c)	If relevant, consider translating estimates of relative risk into absolute risk for a meaningful time period.	Not applicable	Not applicable to this case–control study; ORs are reported as measures of association.
Other analyses	17	Report other analyses performed, e.g., analyses of subgroups and interactions, and sensitivity analyses.	Results, subtype analysis/I-QOL results	Subtype analyses for SUI, UUI, and MUI and I-QOL domain results are reported.
Discussion				
Key results	18	Summarise key results with reference to study objectives.	Discussion, lines 319–388	UI was identified in 687 women (33.3%). Stress urinary incontinence (SUI) was the most common subtype (*n* = 356; 51.8%), followed by urgency urinary incontinence (UUI) (*n* = 191; 27.8%) and mixed urinary incontinence (MUI) (*n* = 140; 20.4%). UI was independently associated with vaginal delivery (OR = 1.48), multiple birth (OR = 2.26), macrosomia (OR = 1.83), and BMI ≥ 25 kg/m^2^ (OR = 2.08). UUI showed the greatest burden, including the lowest total I-QOL score [36.4 (23.9–88.6)].
Limitations	19	Discuss limitations, taking into account sources of potential bias or imprecision. Discuss both direction and magnitude of any potential bias.	Discussion, Limitations lines 430–452	Limitations include medical-facility-based recruitment and potential selection bias, age-only matching with residual confounding by socioeconomic status and parity, questionnaire-based data, and possible inter-assessor variability because several healthcare professionals administered/assessed questionnaires after one-day online training.
Interpretation	20	Give a cautious overall interpretation of results considering objectives, limitations, multiplicity of analyses, results from similar studies, and other relevant evidence.	Discussion	The interpretation focuses on associated factors, subtype-specific quality-of-life differences, limitations of case–control inference, and consistency with previous UI literature.
Generalisability	21	Discuss the generalisability/external validity of the study results.	Discussion, Limitations/Implications	Generalizability to the general female population is limited because participants were recruited through medical settings and specialized UI-care pathways. Findings are most applicable to women attending healthcare institutions in Kazakhstan.
Other information				
Funding	22	Give the source of funding and the role of funders for the present study and, if applicable, the original study on which the article is based.	Funding statement	Not applicable

* Information is presented separately for cases and controls where applicable.

## References

[1] Pizzol D., Demurtas J., Celotto S., Maggi S., Smith L., Angiolelli G., Trott M., Yang L., Veronese N. (2021). Urinary incontinence and quality of life: A systematic review and meta-analysis. Aging Clin. Exp. Res..

[2] Haylen B.T., de Ridder D., Freeman R.M., Swift S.E., Berghmans B., Lee J., Monga A., Petri E., Rizk D.E., Sand P.K. (2010). An International Urogynecological Association (IUGA)/International Continence Society (ICS) joint report on the terminology for female pelvic floor dysfunction. Int. Urogynecol. J..

[3] Milsom I., Gyhagen M. (2019). The prevalence of urinary incontinence. Climacteric.

[4] Wu J.M., Hundley A.F., Fulton R.G., Myers E.R. (2009). Forecasting the prevalence of pelvic floor disorders in U.S. Women: 2010 to 2050. Obstet. Gynecol..

[5] Hunskaar S., Lose G., Sykes D., Voss S. (2004). The prevalence of urinary incontinence in women in four European countries. BJU Int..

[6] Xue K., Palmer M.H., Zhou F. (2020). Prevalence and associated factors of urinary incontinence in women living in China: A literature review. BMC Urol..

[7] Choo M.S., Ku J.H., Oh S.J., Lee K.S., Paick J.S., Seo J.T., Kim D.Y., Lee J.J., Lee J.G., Na Y.G. (2007). Prevalence of urinary incontinence in Korean women: An epidemiologic survey. Int. Urogynecol. J. Pelvic Floor Dysfunct..

[8] Wang Q., Que Y.Z., Wan X.Y., Lin C.Q. (2023). Prevalence, Risk Factors, and Impact on Life of Female Urinary Incontinence: An Epidemiological Survey of 9584 Women in a Region of Southeastern China. Risk Manag. Healthc. Policy.

[9] Abufaraj M., Xu T., Cao C., Siyam A., Isleem U., Massad A., Soria F., Shariat S.F., Sutcliffe S., Yang L. (2021). Prevalence and trends in urinary incontinence among women in the United States, 2005–2018. Am. J. Obstet. Gynecol..

[10] Hammad F.T. (2021). Prevalence, social impact and help-seeking behaviour among women with urinary incontinence in the Gulf countries: A systematic review. Eur. J. Obstet. Gynecol. Reprod. Biol..

[11] Sazonova N.A., Kiseleva M.G., Gadzhieva Z.K., Gvozdev M.Y. (2022). Urinary incontinence in women and its impact on quality of life. Urologiia.

[12] Sharapatov Y., Nurberdiev A., Keulimzhayev N., Botabayeva A., Toleubayev M., Dmitriyeva M., Zhankina R. (2025). Prevalence of Urinary Incontinence and Overactive Bladder Among Female University Students in Kazakhstan. Epidemiologia.

[13] Gaibullaev A.A., Iskandarova G.T., Abdurizaev A.A. (2016). Prevalence and risk factors for urinary incontinence in women living in the South Priaralye region. Urologiia.

[14] Mukhamejan M., Shamshudinov T., Alchinbayev M., Tabynbayev N., Dursun M., Kussainova A., Kassym L., Tsigengagel O., Zhambylov N., Semenova Y. (2026). Long-Term Epidemiological Trends and Regional Disparities in Male Infertility in Central Asia (1991–2023). Int. J. Environ. Res. Public Health.

[15] Bureau of National Statistics of the Agency for Strategic Planning and Reforms of the Republic of Kazakhstan Fertility. https://bala.stat.gov.kz/en/rozhdaemost/.

[16] Republican Center for Health Development Stress Urinary Incontinence in Women. Clinical Protocols of the Ministry of Health of the Republic of Kazakhstan—2024. https://diseases.medelement.com/disease/19037.

[17] National Institute for Health and Care Excellence Urinary Incontinence in Women: Quality Standard QS77. https://www.nice.org.uk/guidance/qs77.

[18] Wacholder S., Silverman D.T., McLaughlin J.K., Mandel J.S. (1992). Selection of controls in case-control studies. II. Types of controls. Am. J. Epidemiol..

[19] Patrick D.L., Martin M.L., Bushnell D.M., Yalcin I., Wagner T.H., Buesching D.P. (1999). Quality of life of women with urinary incontinence: Further development of the incontinence quality of life instrument (I-QOL). Urology.

[20] American College of Obstetricians and Gynecologists (2020). Macrosomia: ACOG Practice Bulletin, Number 216. Obstet. Gynecol..

[21] Royal College of Obstetricians and Gynaecologists A–Z of Medical Terms. https://www.rcog.org.uk/for-the-public/a-z-of-medical-terms/.

[22] Centers for Disease Control and Prevention Adult BMI Categories. https://www.cdc.gov/bmi/adult-calculator/bmi-categories.html.

[23] Davis S.R., Pinkerton J.A., Santoro N., Simoncini T. (2023). Menopause—Biology, consequences, supportive care, and therapeutic options. Cell.

[24] Dietze-Hermosa M., Hitchcock R., Nygaard I.E., Shaw J.M. (2020). Intra-abdominal Pressure and Pelvic Floor Health: Should We Be Thinking About This Relationship Differently?. Female Pelvic Med. Reconstr. Surg..

[25] Yavuz M., Etiler N. (2023). Addressing urinary incontinence by gender: A nationwide population-based study in Turkiye. BMC Urol..

[26] Felippe M.R., Zambon J.P., Girotti M.E., Burti J.S., Hacad C.R., Cadamuro L., Almeida F. (2017). What Is the Real Impact of Urinary Incontinence on Female Sexual Dysfunction? A Case Control Study. Sex. Med..

[27] O’Halloran T., Bell R.J., Robinson P.J., Davis S.R. (2012). Urinary incontinence in young nulligravid women: A cross-sectional analysis. Ann. Intern. Med..

[28] Koli N., Parle D.J., Pardeshi D.T. (2022). A Survey of Urinary Incontinence in Multigravida Females: A Cross-Sectional Study. Int. J. Health Sci. Res..

[29] Rijal C., Hakim S. (2014). Urinary Incontinence in Women Living in Nursing Homes: Prevalence and Risk Factors. Indones. J. Obstet. Gynecol..

[30] Zhang N., He Y., Wang J., Zhang Y., Ding J., Hua K.Q. (2016). Effects of a new community-based reproductive health intervention on knowledge of and attitudes and behaviors toward stress urinary incontinence among young women in Shanghai: A cluster-randomized controlled trial. Int. Urogynecol. J..

[31] Tähtinen R.M., Cartwright R., Tsui J.F., Aaltonen R.L., Aoki Y., Cárdenas J.L., El Dib R., Joronen K.M., Al Juaid S., Kalantan S. (2016). Long-term Impact of Mode of Delivery on Stress Urinary Incontinence and Urgency Urinary Incontinence: A Systematic Review and Meta-analysis. Eur. Urol..

[32] Wesnes S.L., Seim E. (2020). Birthweight and urinary incontinence after childbirth: A systematic review and meta-analysis. Eur. J. Obstet. Gynecol. Reprod. Biol. X.

[33] Zhu J., Si J., Zhao L., Liu W. (2023). Association between infant birthweight and pelvic floor muscle strength: A population-based cohort study. BMC Pregnancy Childbirth.

[34] Progetto Menopausa Italia Study Group (2000). Risk factors for genital prolapse in non-hysterectomized women around menopause. Results from a large cross-sectional study in menopausal clinics in Italy. Eur. J. Obstet. Gynecol. Reprod. Biol..

[35] Wang Q., Manodoro S., Lin H., Li X., Lin C., Jiang X. (2025). Risk Factors and a Predictive Model for Mixed Urinary Incontinence among Parous Women: Insights from a Large-Scale Multicenter Epidemiological Investigation. Digit. Health.

[36] Sun Y., Chen H., Bai Y., Zhang T., Bai W., Jiang B. (2022). Ketogenic diet may be a new approach to treatment stress urinary incontinence in obese elderly women: Report of five cases. BMC Womens Health.

[37] Development Initiatives. Kazakhstan Nutrition Profile. Global Nutrition Report. https://globalnutritionreport.org/resources/nutrition-profiles/asia/central-asia/kazakhstan/.

[38] Weinberg A.E., Leppert J.T., Elliott C.S. (2015). Biochemical Measures of Diabetes are Not Independent Predictors of Urinary Incontinence in Women. J. Urol..

[39] Kwon C.S., Lee J.H. (2014). Prevalence, Risk Factors, Quality of Life, and Health-Care Seeking Behaviors of Female Urinary Incontinence: Results From the 4th Korean National Health and Nutrition Examination Survey VI (2007–2009). Int. Neurourol. J..

[40] Komesu Y.M., Schrader R.M., Ketai L.H., Rogers R.G., Dunivan G.C. (2016). Epidemiology of Mixed, Stress, and Urgency Urinary Incontinence in Mid-Aged/Older Women: The Importance of Incontinence History. Int. Urogynecol. J..

[41] Waetjen L.E., Liao S., Johnson W.O., Sampselle C.M., Sternfield B., Harlow S.D., Gold E.B. (2007). Factors Associated with Prevalent and Incident Urinary Incontinence in a Cohort of Midlife Women: A Longitudinal Analysis of Data from the Study of Women’s Health Across the Nation. Am. J. Epidemiol..

[42] Riss P., Kargl J. (2011). Quality of life and urinary incontinence in women. Maturitas.

[43] Frick A.C., Huang A.J., Van den Eeden S.K., Knight S.K., Creasman J.M., Yang J., Ragins A.I., Thom D.H., Brown J.S. (2009). Mixed urinary incontinence: Greater impact on quality of life. J. Urol..

[44] Minassian V.A., Devore E., Hagan K., Grodstein F. (2013). Severity of urinary incontinence and effect on quality of life in women by incontinence type. Obstet. Gynecol..

[45] Alasmi R.A., Saqyan T.M.B., Alanazi L.F., Alharbi M.F., Alashgae A.F. (2023). Urinary incontinence: Comparison study to identify the type, incidence and risk factors between admitted women and the general population in Al-Kharj city, Saudi Arabia. Urol. Ann..

[46] Alonezy M.F., Metwally A.S., Alhazmi O.A., Alrehaili A.O., Almohammadi A.A., Aljuhani A.S., Alharthi F.A., Aloufi N.A. (2024). The prevalence and related risk factors of urinary incontinence among adult women in Al medina Al Munawara, Saudi Arabia. Cureus.

[47] National Institute for Health and Care Excellence (2021). Pelvic Floor Dysfunction: Prevention and Non-Surgical Management. https://www.nice.org.uk/guidance/ng210.

