# Supplementary material for: Risk Factors and Quality of Life in Women with Urinary Incontinence in Kazakhstan: A Multicenter Case–Control Study
Source: Int J Environ Res Public Health. 2026 Jul 10;23(7):893. doi: 10.3390/ijerph23070893 (PMC13410073; doi:10.3390/ijerph23070893)
Supplement: Supplementary file 1 [file ijerph-23-00893-s001.zip › ijerph-4364112-supplementary.pdf]

**Supplementary Table S1.** Risk factors stratified by the UI subtype

| | **SUI (*n*=356)** | **UUI (*n*=191)** | **MUI (*n*=140)** | ***p*-value** |
|---|---|---|---|---|
| Parity (*n*, %) | | | | |
| 0 | 21 (5.9%) | 18 (9.4%) | 4 (2.9%) | 0.0007 |
| 1–2 | 146 (41.0%) | 69 (36.1%) | 49 (35.0%) | |
| 3–4 | 148 (41.6%) | 84 (44.0%) | 52 (37.1%) | |
| ≥5 | 41 (11.5%) | 20 (10.5%) | 35 (25.0%) | |
| Delivery mode (*n*, %) | | | | |
| Vaginal delivery | 275 (77.2%) | 143 (74.9%) | 113 (80.7%) | 0.311 |
| Cesarean section | 28 (7.9%) | 11 (5.8%) | 11 (7.9%) | |
| Nulliparous (no history of childbirth) | 21 (5.9%) | 18 (9.4%) | 4 (2.9%) | |
| Mixed (vaginal and cesarean deliveries) | 32 (9.0%) | 19 (9.9%) | 12 (8.6%) | |
| Multiple birth (*n*, %) | | | | |
| Yes | 52 (14.6%) | 23 (12.0%) | 18 (12.9%) | 0.149 |
| No | 283 (79.5%) | 150 (78.5%) | 118 (84.3%) | |
| Nulliparous (no history of childbirth) | 21 (5.9%) | 18 (9.4%) | 4 (2.9%) | |
| Macrosomia (*n*, %) | | | | |
| Yes | 61 (17.1%) | 42 (22.0%) | 68 (48.6%) | <0.001 |
| No | 274 (77.0%) | 131 (68.6%) | 68 (48.6%) | |
| Nulliparous (no history of childbirth) | 21 (5.9%) | 18 (9.4%) | 4 (2.9%) | |
| BMI (*n*, %) | | | | |
| <18.5 | 8 (2.2%) | 3 (1.6%) | 1 (0.7%) | <0.001 |
| 18.5–24.9 | 147 (41.3%) | 47 (24.6%) | 31 (22.1%) | |
| 25.0–29.9 | 157 (44.1%) | 99 (51.8%) | 65 (46.4%) | |
| ≥30.0 | 44 (12.4%) | 42 (22.0%) | 43 (30.7%) | |
| Heavy lifting (*n*, %) | | | | |
| Yes | 285 (80.1%) | 165 (86.4%) | 111 (79.3%) | 0.157 |
| No | 62 (17.4%) | 23 (12.0%) | 22 (15.7%) | |
| Not sure | 9 (2.5%) | 3 (1.6%) | 7 (5.0%) | |
| Diabetes (*n*, %) | | | | |
| Yes | 27 (7.6%) | 25 (13.1%) | 17 (12.1%) | 0.005 |
| No | 310 (87.1%) | 164 (85.9%) | 122 (87.1%) | |
| Not sure | 19 (5.3%) | 2 (1.0%) | 1 (0.7%) | |
| Arterial hypertension (*n*, %) | | | | |
| Yes | 109 (30.6%) | 78 (40.8%) | 51 (36.4%) | <0.001 |
| No | 216 (60.7%) | 110 (57.6%) | 86 (61.4%) | |
| Not sure | 31 (8.7%) | 3 (1.6%) | 3 (2.1%) | |
| Pelvic surgery (*n*, %) | | | | |
| Yes | 39 (11.0%) | 32 (16.8%) | 21 (15.0%) | <0.001 |
| No | 317 (89.0%) | 156 (81.7%) | 107 (76.4%) | |
| Not sure | 0 (0.0%) | 3 (1.6%) | 12 (8.6%) | |
| Back pain (*n*, %) | | | | |
| Yes | 78 (21.9%) | 105 (55.0%) | 55 (39.3%) | <0.001 |
| No | 260 (73.0%) | 85 (44.5%) | 83 (59.3%) | |
| Not sure | 18 (5.1%) | 1 (0.5%) | 2 (1.4%) | |
| Menopause (*n*, %) | | | | |
| Yes | 166 (46.6%) | 155 (81.2%) | 95 (67.9%) | <0.001 |
| No | 182 (51.1%) | 33 (17.3%) | 42 (30.0%) | |
| Not sure | 8 (2.2%) | 3 (1.6%) | 3 (2.1%) | |
